# Global, regional, and national burden of gastric cancer attributable to smoking and a high-sodium diet from 1990 to 2021: a global burden of disease study 2021

**DOI:** 10.1186/s12876-025-04431-8

**Published:** 2025-12-03

**Authors:** Guangying Zhao, Yan Liu, Haojie Wang, Yongjian Zhang, Liang Shang, Leping Li

**Affiliations:** 1 Department of Gastrointestinal Surgery, Shandong Provincial Hospital, Affiliated to Shandong First Medical University, Jinan, China; 2 Department of Gastrointestinal Surgery, Shandong Provincial Hospital, Shandong University, Jinan, China

**Keywords:** Gastric cancer, Smoking, High-sodium diet, Global burden of disease, Disability-adjusted life year

## Abstract

**Purpose:**

This study comprehensively assesses the global, regional, and national burden of gastric cancer (GC) attributable to smoking and a high-sodium diet from 1990 to 2021, aiming to promote healthy lifestyle habits and provide scientific evidence for policy-making.

**Methods:**

Data on mortality, disability-adjusted life years (DALYs), the age-standardized mortality rate (ASMR), and the age-standardized DALY rate (ASDR) of GC attributable to smoking and a high-sodium diet were extracted from the Global Burden of Disease (GBD) 2021 study. From 1990 to 2021, the estimated annual percentage change (EAPC) was calculated to clarify the temporal trends in the ASMR and ASDR.

**Results:**

Globally, smoking was responsible for 107,926 (95% UI: 84,603–138,448) deaths from GC in 2021, whereas high sodium intake was associated with 75,661 (95% UI: 0-372,194) deaths. From 1990 to 2021, the number of deaths and DALYs due to GC caused by smoking and a high-sodium diet tended to decrease, with the ASMRs and ASDRs also decreasing. Across all periods and regions, the number of deaths, DALYs, ASMRs, and ASDRs related to smoking and a high-sodium diet were greater in males than in females. East Asia, particularly China, had the highest burden. High- and high-middle-SDI regions experienced decreases, while other regions saw increases.

**Conclusion:**

Despite the global decline in the ASMRs and ASDRs of GC attributable to smoking and a high-sodium diet, the absolute number of gastric cancer deaths caused by a high-sodium diet continues to increase because of population growth and ageing, and significant regional differences persist across age groups and regions. Through a comprehensive analysis on a global scale, this study identified the main modifiable risk factors, providing data support and a theoretical basis for the formulation of risk attribution, stratification, and prevention strategies.

**Supplementary Information:**

The online version contains supplementary material available at 10.1186/s12876-025-04431-8.

## Introduction

 Gastric cancer (GC) is among the most common malignant tumours worldwide, and despite the decline in its incidence and mortality in recent years, it remains among the most common and deadly diseases worldwide [1]. GC ranks 5th in global cancer incidence, with more than 1.23 million new cases, accounting for 8.2% of all cancer-related deaths worldwide, especially in East Asia, where the incidence is highest; and ranks 3rd in global cancer mortality, causing 954,373.60 deaths globally, accounting for 8.2% of cancer-related deaths worldwide [2]. As a highly aggressive tumor, it poses a great threat to the health and life of patients, and at the same time imposes a heavy burden on the global public health system.

Studies have shown that gastric cancer can be caused by a variety of factors, including Helicobacter pylori (Hp) colonization [3], the environment, genetic susceptibility [4], daily behaviours and dietary factors [5]. Daily lifestyle choices, especially excessive salt intake and tobacco use, are modifiable behavioural risk factors that significantly affect cancer outcomes [6, 7]. Specifically, gastric cancer is categorized as a tobacco-related cancer, and smoking is associated with a higher burden of gastric cancer. A systematic review and meta-analysis confirmed cigarette smoking as an independent risk factor for GC, particularly for gastric cardia. The risk of gastric cancer increases with the number of cigarettes smoked and in a dose-dependent manner with respect to smoking duration [8]. In addition, dietary factors, such as alcohol intake, high-sodium diets, diets low in vitamins A and C, and the intake of large amounts of red and processed meat, are strongly associated with an increased risk of gastric cancer [9]. A systematic review and meta-analysis of case‒control studies revealed a significant positive association between high salt intake and gastric cancer compared with low salt intake [10]. The high prevalence of high-sodium diets is associated with a high burden of gastric cancer. In summary, although extensive research has identified smoking and a high-sodium diet as key risk factors for gastric cancer, a thorough analysis of the global, regional, and national burden of gastric cancer mortality and disability-adjusted life years (DALYs) is still notably lacking [11, 12]. Such analyses are critical for identifying at-risk regions and populations and for providing targeted intervention strategies.

The aim of this study is to quantify the burden of GC deaths and disability-adjusted life years (DALYs) attributable to smoking and a high-sodium diet using data from the Global Burden of Disease (GBD) Study 2021. The aim is to provide valuable insights into the impacts of these two modifiable risk factors on global health and identify key areas for intervention—findings that will inform policies and programs aimed at promoting healthy lifestyle habits and reducing the global burden of GC.

## Methods

### Data source

The GBD study, a collaborative initiative led by the Institute for Health Metrics and Evaluation (IHME), aims to identify, compile, and standardize health loss from diseases, injuries, and risk factors across ages, genders, and geographies at specific time points. GBD 2021 covers 204 nations and territories (1990–2021), integrating data from disease registries, household surveys, and demographic censuses to provide age- and sex-stratified estimates for 369 diseases/injuries, 87 risk factors, and key metrics including incidence, prevalence, mortality, years of life lost (YLLs), years lived with disability (YLDs), and DALYs. DALYs, a core GBD metric, are calculated as specified in the IHME/GBD methodology literature [13].

For this study, we extracted 1990–2021 annual data on GC deaths and DALYs attributable to smoking and a high-sodium diet (across 5 sociodemographic index quintiles, 21 GBD regions, and 204 countries/territories) via the Global Health Data Exchange (GHDx) query tool (http://ghdx.healthdata.org/gbd-results-tool, accessed 6 March 2025).

### Case identification and exposure definition

In this study, we targeted the GC burden attributable to the most prominent risk factors, including smoking and a high-sodium diet. Cancerous growth that develops from the stomach’s epithelial tissue is known as GC, and the cancer cells typically originate from the gastric mucosal epithelial cells. According to the tenth revision of the International Classification of Diseases (ICD-10), the code for malignant neoplasms of the stomach is C16. In addition, smoking (excluding second-hand smoke and smokeless tobacco use) is defined as the current daily or occasional use of any smoked tobacco product [14]. A high-sodium diet is defined on the basis of the theoretical minimum risk exposure level (TMREL) of 1–5 g per day [15]; specifically, it refers to an average 24-hour urinary sodium excretion (a biomarker for sodium intake) that exceeds this range [15–17],and its assessment, such as detailed information about the data selection and data inputs, has been described in previous studies.

### The sociodemographic index

The GBD 2021 study employs the sociodemographic index (SDI) as a composite metric for socioeconomic conditions that correlate significantly with health outcomes. The sociodemographic index integrates per capita income, educational attainment, and total fertility rate (TFR) to evaluate a nation’s sociodemographic progress. The GBD 2021 study categorizes the SDI into five levels: high (> 0.810), medium–high (0.713 ~ 0.810), medium (0.620 ~ 0.712), medium–low (0.466 ~ 0.619), and low (< 0.466).

### Statistical analysis

This study utilized point estimates and 95% uncertainty intervals (UIs) for gastric cancer burden—including death counts, DALYs, ASMRs, and ASDRs—attributable to smoking and a high-sodium diet, as provided by the GBD 2021 study [18].

The GBD study generates these estimates through a comprehensive modeling process that propagates uncertainty from all levels of the analysis, including input data, exposure assessment, and relative risks. Point estimates represent the mean of the posterior distribution derived from these models. The 95% UIs are defined by the 2.5th and 97.5th percentiles of this posterior distribution. A key step in the comparative risk assessment framework is the application of a non-negative constraint, which resets any computational draw yielding a negative attributable fraction to zero prior to the final calculation of the UIs.

Temporal trends in the ASMRs and ASDRs of attributable GCs were assessed using the estimated annual percentage change (EAPC). A regression line was fitted to the natural logarithm of the rates, and the EAPC was calculated from the model’s slope. The associated 95% confidence interval for the EAPC was derived from this linear regression model. A trend was considered statistically significant and increasing if both the EAPC value and the lower limit of its 95% CI were greater than 0; conversely, it was considered significant and decreasing if both the EAPC value and the upper limit of its 95% CI were less than 0.

## Results

### Global burden of GC attributable to to smoking and a high-sodium diet in 2021

In 2021, the number of gastric cancer deaths due to smoking was 107,926 (95% UI: 84,603–138,448) globally, with an ASMR of 1.254 (95% UI: 0.981–1.605) per 100,000 population. Specifically, the number of smoking-related GC deaths was 100,322 (95% UI: 78,097–129,830) for males and 7,604 (95% UI: 6,002–9,441) for females, with corresponding ASMRs of 2.562 (95% UI: 1.99–3.298) and 0.163 (95% UI: 0.129–0.203), respectively, per 100,000 population.

In addition, the total number of DALYs associated with the risk of smoking was 2,537,998 (95% UI: 1,991,161–3,270,229), and the DALYs for males and females were 2,373,217 (95% UI: 1,848,952–3,077,861) and 164,781 (95% UI: 132,120–203,386), respectively. The ASDR for smoking-related GC was 29.006 (95% UI: 22.746–37.324) per 100,000 population, with males having an ASDR of 57.567 (95% UI: 44.842–74.594) per 100,000 population and females having an ASDR of 3.588 (95% UI: 2.877–4.421) per 100,000 population (Table 1).

**Table 1:** Number of deaths, ASMRs, DALY numbers, and ASMRs of GC attributable to smoking and a high-sodium diet in 1990 and 2021 and the corresponding temporal trends from 1990 to 2021

Location	Deaths					DALYs				
	1990 Deaths number (95% UI)	1990 ASMR per 100,000 (95% UI)	2021 Deaths number (95% UI)	2021 ASMR per 100,000 (95% UI)	1990-2021 EAPC (95% CI)	1990 DALYs number (95% UI)	1990 ASDR per 100,000 (95% UI)	2021 DALYs number (95% UI)	2021 ASDR per 100,000 (95% UI)	1990-2021 EAPC (95% CI)
**Smoking**										
**Global**	109818(89441–131513)	2.807 (2.286–3.363.286.363)	107926(84603–138448)	1.254 (0.981–1.605.981.605)	−2.63(−2.68 to −2.58)	2929437(2380225–3509272)	71.17 (58.036–85.11.036.11)	2537998(1991161–3270229)	29.006 (22.746–37.324.746.324)	−2.94 (−2.99 to −2.89)
**Male**	99507(80639–120245)	5.656 (4.584–6.844.584.844)	100322(78097–129830)	2.562 (1.99–3.298.99.298)	−2.58 (−2.63 to −2.53)	2685142(2163730–3230878)	138.96 (111.93–167.451.93.451)	2373217(1848952–3077861)	57.567 (44.842–74.594.842.594)	−2.88 (−2.93 to −2.83)
**Female**	10311(8231–12573)	0.501 (0.401–0.613.401.613)	7604(6002-9441)	0.163 (0.129–0.203.129.203)	−3.77 (−3.88 to −3.66)	244295(199026–293805)	11.447 (9.28–13.804.28.804)	164781(132120–203386)	3.588 (2.877–4.421.877.421)	−3.86 (−3.95 to −3.77)
**High SDI**	26991(22570–31941)	2.417 (2.023–2.856.023.856)	16255(13205–19907)	0.734 (0.598–0.891.598.891)	−3.99(−4.04 to −3.93)	629078(530058–735218)	57.902 (48.804–67.696.804.696)	317863(260975–381945)	15.836 (12.986–18.913.986.913)	−4.3(−4.35 to −4.25)
**High-middle SDI**	39723(32105–47502)	3.978 (3.217–4.753.217.753)	39047(30050–51057)	1.95 (1.502–2.548.502.548)	−2.32(−2.4 to −2.24)	1084808(874067–1299890)	105.191 (84.775–126.104.775.104)	925650(712993–1220612)	46.469 (35.851–61.162.851.162)	−2.69(−2.77 to −2.61)
**Middle SDI**	35854(27881–45182)	3.502 (2.719–4.448.719.448)	43073(32395–57603)	1.633 (1.231–2.178.231.178)	−1.6(−1.67 to −1.53)	36731(26058–46343)	90.288 (69.964–113.531.964.531)	46646(31155–58286)	37.476 (28.132–50.226.132.226)	−1.85(−1.92 to −1.78)
**Low-middle SD**	5875(4621-7463)	0.991 (0.782–1.259.782.259)	7784(5978-9586)	0.558 (0.428–0.686.428.686)	−1.77(−1.83 to −1.71)	166305(130651–210207)	25.324 (19.868–32.04.868.04)	205166(157311–253378)	13.64 (10.453–16.825.453.825)	−1.92(−1.97 to −1.87)
**Low SDI**	1287(916–1623)	0.585 (0.417–0.737.417.737)	1710(1127-2129)	0.357 (0.233–0.445.233.445)	−2.45(−2.52 to −2.37)	1010299(774014–1270169)	14.987 (10.67–18.911.67.911)	1041308(777836–1396036)	8.645 (5.72–10.792.72.792)	−2.84(−2.9 to −2.77)
**East Asia**	52449(38960–66990)	6.111 (4.581–7.851.581.851)	66779(49132–92419)	3.044 (2.245–4.209.245.209)	−2.17(−2.29 to −2.05)	1475263(1091203–1885797)	157.069(116.543–200.353.543.353)	1580210(1162456–2205354)	70.004 (51.492–97.495.492.495)	−2.57(−2.67 to −2.48)
**Southeast Asia**	2897(2141-3628)	1.177 (0.873–1.48.873.48)	3962(3093-5118)	0.613 (0.482–0.786.482.786)	−2.38(−2.48 to −2.29)	80944(59428–101197)	29.629 (21.83–37.04.83.04)	107993(83679–138316)	15.306 (11.901–19.745.901.745)	−2.37(−2.45 to −2.29)
**Central Asia**	1231(1024-1478)	2.563 (2.124–3.087.124.087)	933(752–1128)	1.141 (0.918–1.384.918.384)	−2.13(−2.3 to −1.97)	36381(30565–43166)	72.783 (61.03–86.716.03.716)	26008(21089–31451)	29.302 (23.737–35.43.737.43)	−2.56(−2.7 to −2.43)
**High-income Asia Pacific**	11774(9706–14249)	5.916 (4.882–7.167.882.167)	7130(5654-9036)	1.361 (1.1–1.683.1.683)	−4.98(−5.06 to −4.89)	287266(235854–343553)	139.685(115.134–167.027.134.027)	124539(100751–153825)	28.051 (23.136–33.92.136.92)	−5.38(−5.47 to −5.3)
**Eastern Europe**	9199(7745–10702)	3.225 (2.712–3.756.712.756)	4418(3628-5314)	1.256 (1.03–1.504.03.504)	−3.29(−3.49 to −3.1)	265795(224633–306469)	93.578 (79.178–108.116.178.116)	115871(95233–138251)	34.146 (28.071–40.608.071.608)	−3.55(−3.74 to −3.36)
**Oceania**	37(25–50)	1.206 (0.837–1.622.837.622)	60(43–84)	0.75 (0.536–1.02.536.02)	−1.66(−1.76 to −1.57)	1179(776–1628)	33.568 (22.788–45.782.788.782)	1976(1375-2757)	21.349 (15.003–29.598.003.598)	−1.58(−1.69 to −1.48)
**Central Europe**	3778(3190-4426)	2.508 (2.115–2.946.115.946)	2110(1701-2539)	0.954 (0.773–1.146.773.146)	−3.11(−3.23 to −2.99)	36381(30565–43166)	64.062 (54.292–74.995.292.995)	26008(21089–31451)	23.936 (19.575–28.657.575.657)	−3.16(−3.27 to −3.04)
**Australasia**	190(152–231)	0.799 (0.639–0.97.639.97)	129(100–166)	0.238 (0.187–0.303.187.303)	−3.91(−4.03 to −3.78)	4426(3586-5262)	18.886 (15.344–22.415.344.415)	2754(2199-3422)	5.656 (4.548–6.958.548.958)	−3.88(−4.01 to −3.76)
**Western Europe**	12415(10171–14686)	2.083 (1.713–2.459.713.459)	5545(4382-6788)	0.569 (0.456–0.688.456.688)	−4.15(−4.2 to −4.1)	262440(216926–307176)	46.422 (38.48–54.257.48.257)	108740(87991–130445)	12.747 (10.429–15.256.429.256)	−4.09(−4.13 to −4.05)
**Southern Latin America**	764(615–918)	1.637 (1.321–1.976.321.976)	651(521–797)	0.754 (0.606–0.921.606.921)	−2.37(−2.53 to −2.2)	20744(16856–24556)	44.13 (35.885–52.257.885.257)	16801(13645–20302)	20.036 (16.328–24.229.328.229)	−2.42(−2.59 to −2.26)
**High-income North America**	3432(2774-4132)	0.963 (0.78–1.158.78.158)	2380(1846-2970)	0.355 (0.278–0.442.278.442)	−3.31(−3.36 to −3.26)	78133(63544–93566)	22.944 (18.656–27.423.656.423)	51015(40682–62404)	8.161 (6.581–9.935.581.935)	−3.4(−3.45 to −3.35)
**Caribbean**	242(196–295)	0.958 (0.772–1.174.772.174)	260(199–322)	0.48 (0.368–0.595.368.595)	−2.23(−2.31 to −2.15)	5919(4816-7124)	22.597 (18.356–27.239.356.239)	6247(4800-7656)	11.567 (8.892–14.19.892.19)	−2.19(−2.27 to −2.11)
**Andean Latin America**	277(223–346)	1.405 (1.122–1.753.122.753)	439(332–581)	0.754 (0.569–1.569)	−2.12(−2.26 to −1.98)	7132(5715-8836)	33.952 (27.271–42.339.271.339)	10674(7937–14162)	17.798 (13.24–23.646.24.646)	−2.2(−2.33 to −2.06)
**Central Latin America**	1107(909–1308)	1.413 (1.155–1.682.155.682)	1160(916–1439)	0.467 (0.369–0.579.369.579)	−3.95(−4.09 to −3.8)	28564(23674–33643)	33.142 (27.391–39.148.391.148)	29382(23108–36220)	11.443 (9.021–14.132.021.132)	−3.81(−3.95 to −3.67)
**Tropical Latin America**	2465(1989-2983)	2.853 (2.286–3.492.286.492)	1890(1502-2351)	0.741 (0.587–0.925.587.925)	−4.59(−4.71 to −4.48)	63812(52072–75933)	67.352 (54.741–80.784.741.784)	45162(36443–55400)	17.285 (13.934–21.212.934.212)	−4.69(−4.81 to −4.56)
**North Africa and Middle East**	2674(1855-3426)	1.677 (1.169–2.145.169.145)	3703(2333-4614)	0.889 (0.56–1.112.56.112)	−2.01(−2.09 to −1.94)	72912(50364–93115)	40.823 (28.282–52.385.282.385)	93116(59062–116916)	19.84 (12.532–24.809.532.809)	−2.33(−2.41 to −2.25)
**Central Sub-Saharan Africa**	96(62–127)	0.418 (0.27–0.547.27.547)	145(95–197)	0.255 (0.168–0.343.168.343)	−1.56(−1.75 to −1.36)	2874(1864-3833)	11.215 (7.242–14.839.242.839)	4479(2918-6072)	6.883 (4.511–9.345.511.345)	−1.54(−1.72 to −1.36)
**South Asia**	4119(3146-5417)	0.73 (0.558–0.957.558.957)	5370(4033-6893)	0.376 (0.282–0.481.282.481)	−2.04(−2.13 to −1.95)	118993(90634–156081)	18.912 (14.439–24.876.439.876)	138736(105641–179492)	9.041 (6.856–11.689.856.689)	−2.29(−2.38 to −2.21)
**Southern Sub-Saharan Africa**	190(137–235)	0.709 (0.513–0.889.513.889)	226(175–278)	0.383 (0.294–0.471.294.471)	−2.19(−2.45 to −1.93)	5560(4013-6903)	18.834 (13.53–23.4.53.4)	6702(5193-8214)	10.463 (8.066–12.849.066.849)	−2.05(−2.29 to −1.81)
**Eastern Sub-Saharan Africa**	305(215–391)	0.425 (0.301–0.544.301.544)	363(269–457)	0.223 (0.166–0.28.166.28)	−2.31(−2.39 to −2.22)	8665(6032–11066)	10.758 (7.581–13.731.581.731)	10380(7635–13169)	5.591 (4.125–7.041.125.041)	−2.34(−2.42 to −2.25)
**Western Sub-Saharan Africa**	176(133–226)	0.205 (0.156–0.264.156.264)	272(191–348)	0.142 (0.102–0.182.102.182)	−1.01(−1.08 to −0.95)	4955(3713-6284)	5.257 (3.957–6.709.957.709)	7687(5400-9821)	3.545 (2.499–4.529.499.529)	−1.13(−1.2 to −1.06)
**High-sodium diet**										
**Global**	67845(0–339513)	1.744 (0–8.742.742)	75661(0–372194)	0.887 (0–4.37.37)	−2.26 (−2.35 to −2.18)	1845617(0–9206158)	44.532 (0–222.305.305)	1804592(0–8884379)	20.783 (0–102.378.378)	−2.56 (−2.64 to −2.47)
**Male**	43642(0–220825)	2.458 (0–12.425.425)	50374(0–247168)	1.292 (0–6.341.341)	−2.12 (−2.21 to −2.02)	1221058(0–6151893)	62.201 (0–314.711.711)	1231290(0–6026424)	29.9 (0–146.648.648)	−2.42 (−2.52 to −2.33)
**Female**	24202(0–123378)	1.154 (0–5.864.864)	25287(0–129118)	0.547 (0–2.795.795)	−2.57 (−2.67 to −2.48)	624558(0–3176862)	28.715 (0–146.181.181)	573301(0–2940974)	12.607 (0–64.624.624)	−2.84 (−2.93 to −2.74)
**High SDI**	13706(0–69403)	1.238 (0–6.268.268)	12209(0–61722)	0.543 (0–2.733.733)	−2.72(−2.75 to −2.7)	321496(0–1625678)	29.889 (0–151.091.091)	230574(0–1154960)	11.603 (0–58.092.092)	−3.11(−3.13 to −3.08)
**High-middle SDI**	24048(0–122047)	2.442 (0–12.408.408)	23439(0–114553)	1.184 (0–5.791.791)	−2.43(−2.56 to −2.31)	653552(0–3320830)	63.6 (0–322.965.965)	551396(0–2681101)	28.139 (0–136.923.923)	−2.76(−2.9 to −2.63)
**Middle SDI**	23250(0–115303)	2.301 (0–11.483.483)	28816(0–141923)	1.107 (0–5.43.43)	−1.14(−1.19 to −1.08)	667339(0–3270306)	59.025 (0–290.914.914)	712582(0–3527047)	25.818 (0–127.576.576)	−1.41(−1.47 to −1.36)
**Low-middle SD**	4881(0–24751)	0.816 (0–4.115.115)	8255(0–41816)	0.592 (0–3.004.004)	−0.95(−1 to −0.91)	145053(0–737564)	21.392 (0–108.457.457)	226298(0–1148265)	14.783 (0–75.093.093)	−1.13(−1.16 to −1.09)
**Low SDI**	1904(0–9916)	0.858 (0–4.476.476)	2894(0–15443)	0.599 (0–3.183.183)	−2.47(−2.6 to −2.35)	56794(0–295505)	22.466 (0–116.971.971)	82603(0–441281)	14.711 (0–78.407.407)	−2.79(−2.91 to −2.68)
**East Asia**	31816(0–155224)	3.775 (0–18.417.417)	37862(0–188112)	1.763 (0–8.69.69)	−2.54(−2.74 to −2.34)	910166(0–4421005)	96.583 (0–469.067.067)	906420(0–4574158)	41.092 (0–206.627.627)	−2.88(−3.06 to −2.7)
**Southeast Asia**	2264(0–11385)	0.906 (0–4.606.606)	3572(0–18178)	0.564 (0–2.889.889)	−1.71(−1.78 to −1.65)	66676(0–336540)	23.489 (0–118.136.136)	97670(0–497997)	13.981 (0–71.253.253)	−1.85(−1.91 to −1.78)
**Central Asia**	998(0–4972)	1.536 (0–7.688.688)	723(0–3725)	0.712 (0–3.491.491)	−2.54(−2.64 to −2.44)	28790(0–143224)	57.97 (0–288.431.431)	20435(0–105882)	23.075 (0–119.442.442)	−2.8(−2.88 to −2.73)
**High-income Asia Pacific**	6059(0–29937)	3.087 (0–15.256.256)	5864(0–29532)	1.088 (0–5.431.431)	−3.44(−3.48 to −3.39)	151938(0–743670)	74.625 (0–365.657.657)	99017(0–495285)	22.571 (0–112.133.133)	−3.92(−3.97 to −3.87)
**Eastern Europe**	6323(0–32996)	2.248 (0–11.721.721)	3241(0–16645)	0.923 (0–4.736.736)	−3.11(−3.21 to −3.01)	173733(0–903204)	61.634 (0–320.718.718)	79240(0–402533)	23.544 (0–119.384.384)	−3.4(−3.52 to −3.28)
**Oceania**	36(0–196)	1.36 (0–7.124.124)	72(0–381)	1.059 (0–5.52.52)	−0.83(−0.89 to −0.78)	1065(0–5908)	32.706 (0–178.434.434)	2144(0–11558)	25.52 (0–134.982.982)	−0.82(−0.89 to −0.76)
**Central Europe**	2259(0–11339)	1.536 (0–7.688.688)	1600(0–7858)	0.712 (0–3.491.491)	−2.58(−2.67 to −2.5)	55099(0–277909)	36.716 (0–185.314.314)	34860(0–170909)	16.708 (0–81.881.881)	−2.62(−2.71 to −2.54)
**Australasia**	113(0–630)	0.485 (0–2.7.7)	138(0–788)	0.251 (0–1.41.41)	−2.1(−2.22 to −1.99)	2603(0–14210)	11.255 (0–61.373.373)	2809(0–15483)	5.716 (0–31.14.14)	−2.16(−2.26 to −2.05)
**Western Europe**	6132(0–32392)	1.037 (0–5.47.47)	4050(0–21331)	0.406 (0–2.103.103)	−3(−3.1 to −2.9)	127536(0–667811)	22.85 (0–119.244.244)	76171(0–392855)	8.935 (0–45.979.979)	−2.96(−3.05 to −2.88)
**Southern Latin America**	685(0–3455)	1.516 (0–7.605.605)	752(0–3767)	0.85 (0–4.263.263)	−1.69(−1.8 to −1.58)	16278(0–82265)	35.002 (0–176.906.906)	16599(0–82996)	19.426 (0–97.041.041)	−1.73(−1.84 to −1.62)
**High-income North America**	1414(0–7466)	0.398 (0–2.099.099)	1482(0–7675)	0.227 (0–1.172.172)	−1.88(−1.92 to −1.83)	31635(0–165388)	9.344 (0–48.844.844)	32631(0–166471)	5.522 (0–28.105.105)	−1.73(−1.77 to −1.68)
**Caribbean**	236(0–1223)	0.937 (0–4.831.831)	311(0–1671)	0.578 (0–3.099.099)	−1.48(−1.55 to −1.4)	5805(0–30338)	21.926 (0–114.626.626)	7608(0–40990)	14.237 (0–76.674.674)	−1.31(−1.41 to −1.21)
**Andean Latin America**	522(0–2645)	2.669 (0–13.48.48)	988(0–4999)	1.707 (0–8.649.649)	−1.64(−1.79 to −1.5)	13410(0–68084)	62.373 (0–316.563.563)	23128(0–116684)	38.451 (0–193.858.858)	−1.79(−1.93 to −1.64)
**Central Latin America**	1316(0–6625)	1.697 (0–8.527.527)	2295(0–11895)	0.933 (0–4.832.832)	−2.18(−2.26 to −2.1)	33796(0–170528)	38.562 (0–194.235.235)	57297(0–297890)	22.411 (0–116.518.518)	−2(−2.09 to −1.92)
**Tropical Latin America**	1356(0–6802)	1.582 (0–7.948.948)	1988(0–10206)	0.782 (0–4.012.012)	−2.36(−2.42 to −2.31)	35940(0–179652)	37.332 (0–186.563.563)	49223(0–252502)	18.881 (0–96.829.829)	−2.33(−2.39 to −2.27)
**North Africa and Middle East**	1257(0–7341)	0.751 (0–4.399.399)	1977(0–11966)	0.451 (0–2.751.751)	−1.58(−1.64 to −1.51)	37024(0–214621)	19.656 (0–114.766.766)	54759(0–325676)	11.002 (0–65.918.918)	−1.83(−1.9 to −1.77)
**Central Sub-Saharan Africa**	142(0–838)	0.681 (0–4.026.026)	262(0–1544)	0.513 (0–2.992.992)	−0.96(−1 to −0.92)	4237(0–24980)	16.968 (0–100.017.017)	7864(0–46176)	12.538 (0–73.888.888)	−1.02(−1.06 to −0.98)
**South Asia**	3631(0–18683)	0.621 (0–3.207.207)	6499(0–32700)	0.447 (0–2.252.252)	−0.95(−1.03 to −0.86)	112571(0–573788)	17.015 (0–87.294.294)	180806(0–908078)	11.449 (0–57.604.604)	−1.19(−1.26 to −1.11)
**Southern Sub-Saharan Africa**	148(0–796)	0.552 (0–3.003.003)	255(0–1387)	0.45 (0–2.475.475)	−0.69(−1.01 to −0.37)	4431(0–23681)	14.616 (0–78.111.111)	7340(0–39115)	11.519 (0–62.182.182)	−0.75(−1.08 to −0.43)
**Eastern Sub-Saharan Africa**	658(0–3325)	0.895 (0–4.504.504)	852(0–4457)	0.538 (0–2.793.793)	−1.9(−1.99 to −1.81)	19716(0–99793)	23.367 (0–118.118.118)	24470(0–129515)	12.943 (0–67.732.732)	−2.21(−2.32 to −2.11)
**Western Sub-Saharan Africa**	479(0–2559)	0.574 (0–3.062.062)	880(0–4609)	0.488 (0–2.544.544)	−0.28(−0.37 to −0.2)	13169(0–70651)	14.022 (0–75.085.085)	24102(0–126886)	11.316 (0–59.283.283)	−0.48(−0.55 to −0.4)

ith respect to the global burden of gastric cancer (GC) caused by high-sodium diets, the attributable number of deaths in 2021 was 75,661 (95% uncertainty interval [UI]: 0–372,194), with an attributable age-standardized mortality rate (ASMR) of 0.887 (95% UI: 0–4.37) per 100,000 population; the number of deaths was 50,374 (95% UI: 0–247,168) in men and 25,287 (95% UI: 0–129,118) in women, with corresponding ASMRs per 100,000 population of 1.292 (95% UI: 0–6.341) and 0.547 (95% UI: 0–2.795), respectively. Notably, the 95% UIs for these high-sodium diet-attributable metrics span from 0 to substantially high values, indicating relatively unstable model estimates—this may stem from limitations in the GBD study’s risk attribution model for dietary factors or variations in data quality. Thus, the absolute burden values should be interpreted with caution, and greater emphasis should be placed on relative trends (e.g., regional differences in the direction of burden changes) rather than absolute numerical comparisons. Thus, the absolute burden values should be interpreted with caution, and greater emphasis should be placed on relative trends (e.g., regional differences in the direction of burden changes) rather than absolute numerical comparisons. The total number of attributable DALYs was 1,804,592 (95% UI: 0–8,884,379); the quantified DALYs were 1,231,290 (95% UI: 0–6,026,424) in men and 573,301 (95% UI: 0–2,940,974) in women, with corresponding ASDRs of 29.9 (95% UI: 0–146.648) per 100,000 population in males and 12.607 (95% UI: 0–64.624) per 100,000 population in females (Table 1).

### Global burden of GC attributed to smoking and a high-sodium diet from 1990 to 2021

**Fig. 1:**
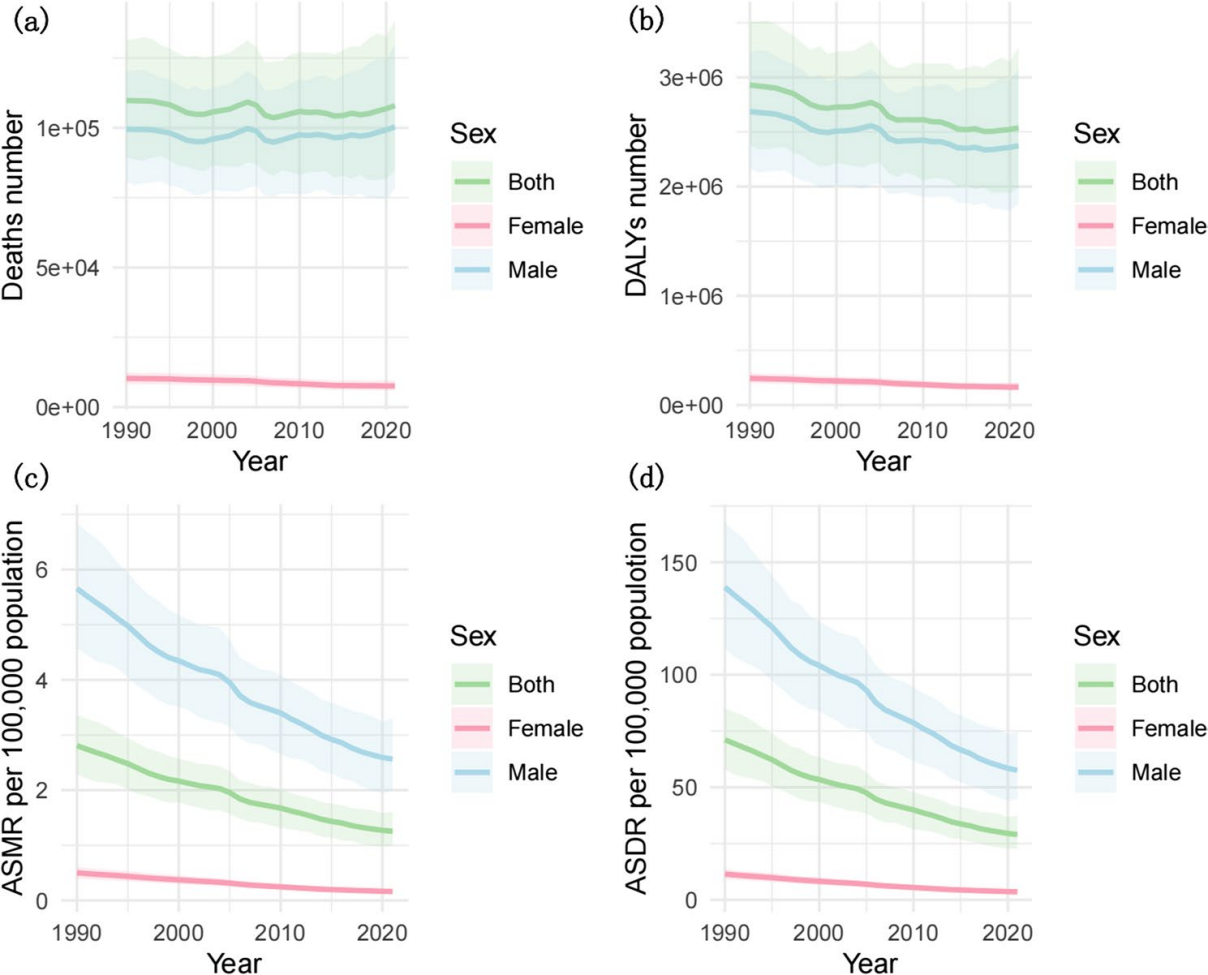
The number of deaths (**a**), DALYs (**b**), ASMRs per 100,000 population (**c**), ASDRs per 100,000 population (**d**) of gastric cancer attributable to smoking by sex, 1990–2021. ASMRs = age-standardised mortality rate; ASDRs = age-standardised DALYs rate; DALYs = disability-adjusted life years

Globally, from 1990 to 2021, the numbers of deaths and DALYs due to GC caused by smoking tended to decrease, and the ASMR and ASDR also decreased; the EAPC values were − 2.63 (95% CI: −2.68 to − 2.58) for deaths and − 2.94 (95% CI: −2.99 to − 2.89) for DALYs. These declining rates were observed in both sexes, with EAPCs in the ASMRs of − 2.58 (95% CI: −2.63 to − 2.53) in men and − 3.77 (95% CI: −3.88 to − 3.66) in women and EAPCs in the ASDRs of − 2.88 (95% CI: −2.93 to − 2.83) in men and − 3.24 (95% CI: −3.36 to − 3.11) in women (Table 1). The global GC trends in deaths, DALYs, ASMRs, and ASDRs attributed to smoking from 1990 to 2021 are shown in Fig. 1. A gradual decline in deaths and DALYs for both males and females is evident, with male figures consistently surpassing those of females (Fig. 1a and b). Both the ASMRs and the ASDRs tended to decrease, with males demonstrating significantly higher rates compared with females (Fig. 1c and d).

**Fig. 2:**
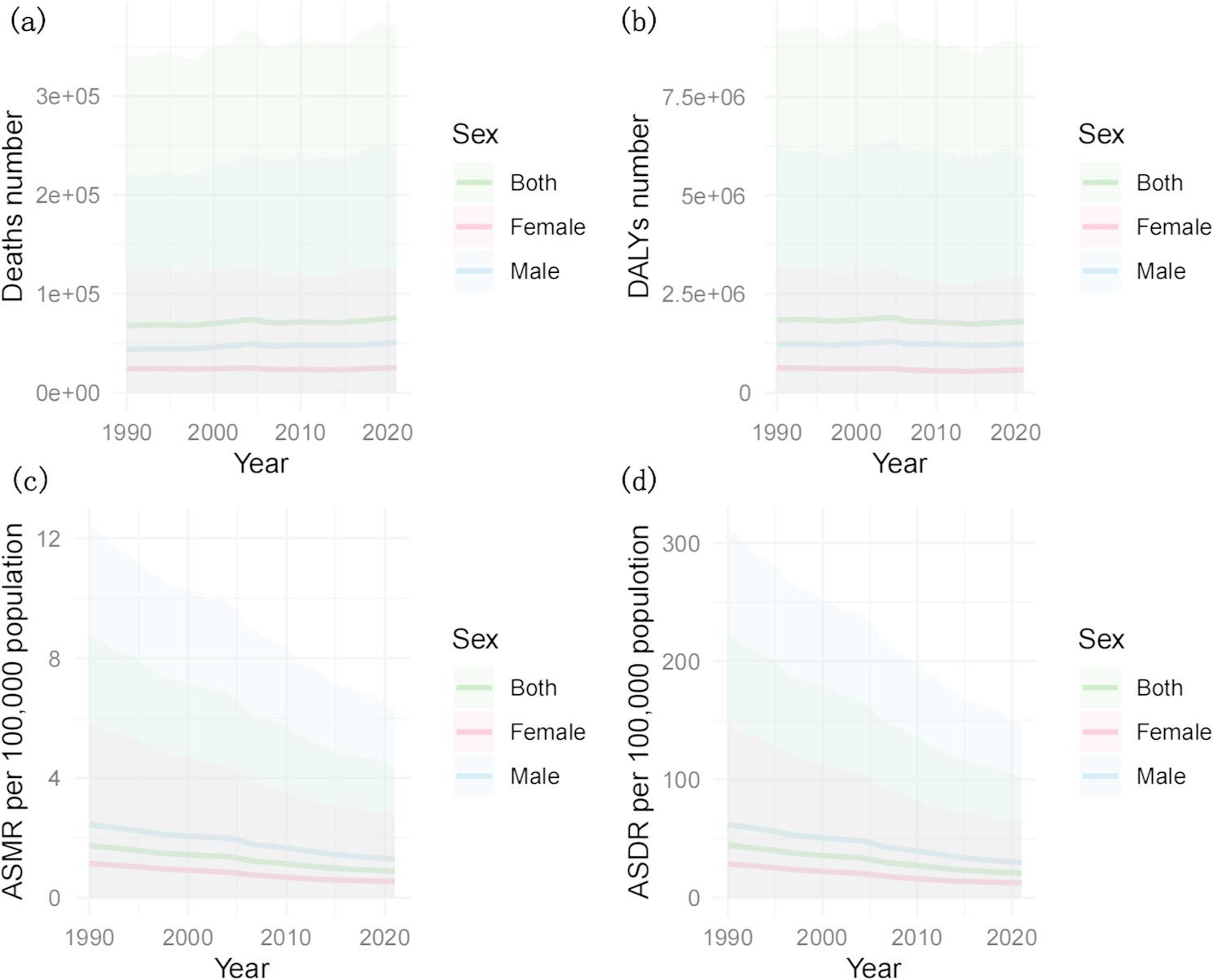
The number of deaths (**a**), DALYs (**b**), ASMRs per 100,000 population (**c**), ASDRs per 100,000 population (**d**) of gastric cancer attributable to a high-sodium diet by sex, 1990–2021

However, the trend of GC estimates related to a high-sodium diet differed. The global attributable death count tended to increase, whereas age-standardized rates decreased, with EAPCs of − 2.26 (95% CI: −2.35 to − 2.18) for the ASMR and − 2.56 (95% CI: −2.64 to − 2.47) for the ASDR. Among males, the EAPCs in ASMR and ASDR were − 2.12 (95% CI: −2.21 to − 2.02) and − 2.42 (95% CI: −2.52 to − 2.33), respectively; among females, the corresponding values were − 2.57 (95% CI: −2.67 to − 2.48) and − 2.84 (95% CI: −2.93 to − 2.74), respectively. Despite these fluctuations, the estimated numbers of GC deaths and DALYs linked to high sodium intake remained stable, with consistently higher attributable values in males than in females; both the ASMR and the ASDR tended to decrease (Fig. 2c and d).

### Regional and National GC burden due to smoking and a high-sodium diet

In the GBD study, East Asia was identified as having the greatest number of deaths due to smoking-related GC in 2021, with a total of 66,779 (95% UI: 49,132–92,419) among the 21 geographically classified regions. From 1990 to 2021, the number of deaths declined in only nine regions: Central Asia, high-income Asia Pacific, Eastern Europe, Central Europe, Australasia, Western Europe, southern Latin America, high-income North America, and tropical Latin America. East Asia had the highest ASMR [3.044 (95% UI: 2.245–4.209)] per 100,000 population in 2021, while Western Sub-Saharan Africa had the lowest ASMR [0.142 (95% UI: 0.102–0.182)] per 100,000 population. Between 1990 and 2021, the EAPC of the ASMRs for GC attributed to smoking decreased in all 21 regions. East Asia also had the highest number of DALYs [1,580,210 (95% UI: 1,162,456–2,205,354)], while Oceania had the lowest number of DALYs [1,976 (95% UI: 1,375–2,757)] in 2021. Furthermore, East Asia had the highest ASDR [70.004 (95% UI: 51.492–97.495)] per 100,000 population in 2021, whereas Western Sub-Saharan Africa had the lowest ASDR [3.545 (95% UI: 2.499–4.529)] per 100,000 population. From 1990 to 2021, both the ASDR and the EAPC tended to decrease in 21 regions (Table 1).

East Asia had the highest attributable death count with respect to a high-sodium diet, at 37,862 (95% UI: 0–188,112), whereas Oceania had the lowest, at 72 (95% UI: 0–381). East Asia also had the highest attributable ASMR, at 1.763 (95% UI: 0–8.69) per 100,000 population, followed by Andean Latin America at 1.707 (95% UI: 0–8.649) per 100,000 population and high-income Asia Pacific at 1.088 (95% UI: 0–5.431) per 100,000 population; high-income North America had the lowest ASMR, at 0.227 (95% UI: 0–1.172) per 100,000 population in 2021. The EAPC in the ASMR decreased across all 21 regions from 1990 to 2021. East Asia had the highest DALYs, at 906,420 (95% UI: 0–4,574,158) per 100,000 population, and together with Andean Latin America, it exhibited the highest ASDRs, at 41.092 (95% UI: 0–206.627) per 100,000 population and 38.451 (95% UI: 0–193.858) per 100,000 population, respectively. ASDR decreased in all 21 regions between 1990 and 2021; the greatest reductions were observed in the high-income Asia Pacific, with an EAPC of − 3.92 (95% CI: −3.97 to − 3.87) per 100,000 population, and in East Asia, with an EAPC of − 2.88 (95% CI: −3.06 to − 2.70) per 100,000 population (Table 1).

**Fig. 3:**
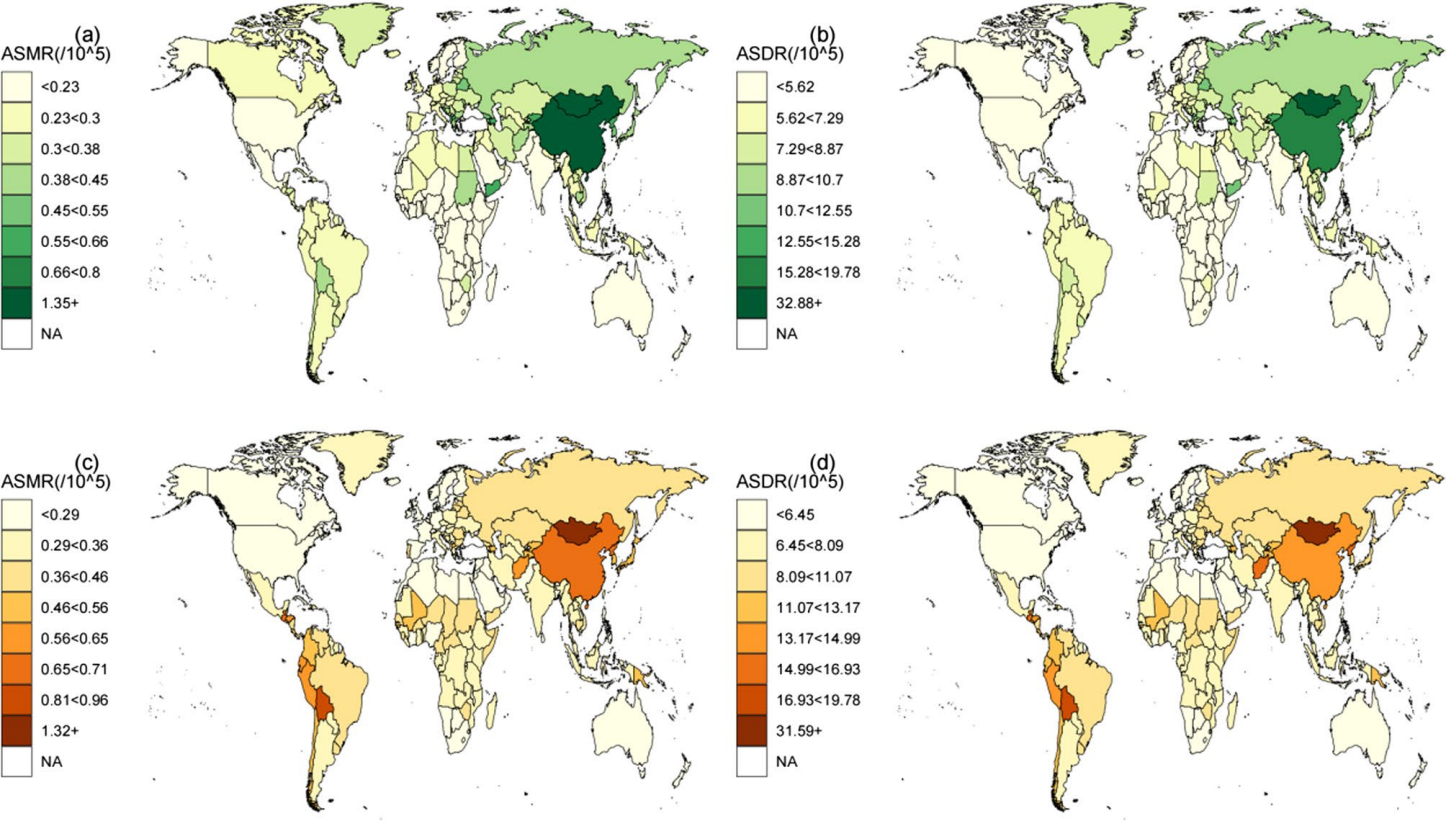
Global distribution of gastric-cancer burden attributable to smoking [(**a**) ASMRs, (**b**) ASDRs] and to a high-sodium diet [(**c**) ASMRs, (**d**) ASDRs], 2021. ASMRs = age-standardized mortality rate; ASDRs = age-standardized DALY rate; DALYs = disability-adjusted life year

In 2021, at the national level, China had the greatest number of GC deaths and DALYs associated with smoking (65,604; 95% UI: 48,081–90,844), followed by Japan (5,440; 95% UI: 4,225–6,938) and India (4,159; 95% UI: 3,107–5,508). China also had the highest number of DALYs (1,550,245; 95% UI: 1,135,747–2,166,216), followed by India (107,766; 95% UI: 80,807–144,331) and Japan (91,630; 95% UI: 73,016–113,321). Mongolia had the highest ASMR of 3.417 (95% UI: 2.443–4.493) per 100,000 population and an ASDR of 92.885 (95% UI: 66.289–126.458) per 100,000 population of GC attributable to smoking, whereas Singapore had the lowest ASMR of −6.04 (95% CI: −6.29 to −5.79) per 100,000 population and an ASDR of −6.27 (95% CI: −6.52 to −6.02) per 100,000 population (Fig. 3a and b, Tables S1 and S2). Between 1990 and 2021, the EAPC of the ASMRs increased in only six countries (Egypt, Georgia, Guinea-Bissau, Honduras, Lesotho, Mali), while the rest showed a downwards trend. The percentage changes in the fraction of all GC deaths and DALYs attributable to smoking are shown in Tables S1 and S2.

In comparison, in 2021, China had the greatest number of GC deaths and DALYs linked to a high-sodium diet: 4,801 (95% UI: 0–24,191) and 883,435 (95% UI: 0–4,461,211), respectively, followed by India with 5,368 (95% UI: 0–27,164) deaths and 149,555 (95% UI: 0–754,254) DALYs and Japan with 4,801 (95% UI: 0–24,191) deaths and 76,537 (95% UI: 0–383,664) DALYs. In nine countries and regions (Greenland, Mariana Islands, Marshall Islands, Nauru, Niue, Palau, Saint Kitts and Nevis, Tokelau, Tuvalu), the number of deaths from GC attributed to a high-sodium diet was less than one. Bolivia, Korea, and China exhibited the highest ASMRs due to a sodium-rich diet, at 1.292 (95% UI: 0–6.341), 1.176 (95% UI: 0–5.924), and 1.088 (95% UI: 0–5.431) per 100,000 population, respectively, whereas Mongolia, Bolivia, and Korea recorded the highest ASDRs, at 29.9 (95% UI: 0–146.648), 28.7 (95% UI: 0–143.5), and 27.8 (95% UI: 0–139.2) per 100,000 population, respectively. Egypt exhibited the most significant increases in the ASMR and ASDR, with EAPCs of 2.49 (95% CI: 1.88–3.10) and 2.07 (95% CI: 1.52–2.63), respectively. During the same period, the ASMRs and ASDRs of Korea decreased most rapidly, with EAPCs of −4.99 (95% CI: −5.14 to −4.84) and − 5.43 (95% CI: −5.55 to −5.30), respectively (Fig. 3c and d, Tables S3 and S4). Additionally, Tables S3 and S4 present the percentage changes in the proportions of all GC deaths and DALYs associated with smoking and a high-sodium diet.

### Global burden of GC attributable to smoking and a high-sodium diet, categorized by sex and age

**Fig. 4:**
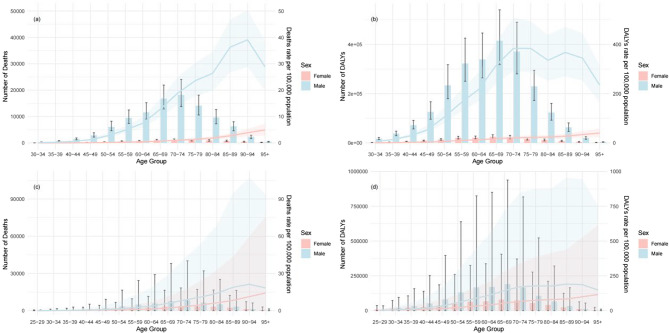
Global deaths and ASMRs (**a**) and global DALYs and ASDRs (**b**) per 100,000 population for gastric cancer attributable to smoking, and global deaths and ASMRs (**c**) and global DALYs and ASDRs (**d**) per 100,000 population for gastric cancer attributable to a high-sodium diet by age and sex in 2021

As depicted in Fig. 4, the numbers of gastric cancer deaths and DALYs attributed to smoking and diets high in sodium are significantly greater in males than in females. Among both males and females, the number of deaths attributable to smoking and a high-sodium diet increased with age, peaking in the 70–74 age group before declining thereafter (Fig. 4a and c). Similarly, DALYs attributed to these risk factors also increased with advancing age, reaching their peak in the 65–69 age group before decreasing (Fig. 4b and d).

In males, the ASMRs of gastric cancer associated with smoking and a high-sodium diet increased with age, peaking at 90–94 years, followed by a decline (Fig. 4a and c). The ASDR of GC attributed to smoking peaked at 70–74 years and subsequently decreased, except for that of the 80–84 age group, and the ASDR of GC attributed to a high-sodium diet peaked at 85–89 years and then declined. In females, the age-specific ratio of death and DALYs consistently increased with age (Fig. 4b and d).

### Association with the SDI

In the high-SDI and high-middle-SDI regions, there were decreases in the numbers of deaths and DALYs attributed to smoking, whereas increases were observed in the remaining three SDI regions. In 2021, middle-SDI regions had the greatest number of deaths—43,073 (95% UI: 32,395–57,603) per 100,000 population—and low-SDI regions had the greatest number of DALYs—1,041,308 (95% UI: 777,836–1,396,036) per 100,000 population. Regions with high-middle-SDI values presented the highest ASMR of 1.95 (95% UI: 1.502–2.548) per 100,000 population, and the highest ASDR: 46.469 (95% UI: 35.851–61.162) per 100,000 population. Conversely, low-SDI regions had the lowest number of deaths—1,710 (95% UI: 1,127–2,129) per 100,000 population—ASMR, with 0.357 (95% UI: 0.233–0.445) per 100,000 population, and ASDR, with 8.645 (95% UI: 5.72–10.792) per 100,000 population; middle-SDI regions had the lowest DALYs: 46,646 (95% UI: 31,155–58,286) per 100,000 population. The lowest EAPC of ASMR, −3.99 (95% CI: −4.04 to −3.93), and the lowest ASDR, −4.3 (95% CI: −4.35 to −4.25), of GC attributed to smoking were observed in the high-SDI region, with the EAPC declining in all five SDI regions over the period from 1990 to 2021 (Table 1).

In 2021, the highest numbers of GC deaths and DALYs attributed to a high-sodium diet were observed in the middle-SDI regions, at 28,816 (95% UI: 0–141,923) deaths and 712,582 (95% UI: 0–3,527,047) DALYs, respectively. Regions with high-middle-SDI values had the highest attributable ASMRs—1.184 (95% UI: 0–5.791) per 100,000 population—and ASDR, with 28.139 (95% UI: 0–136.923) per 100,000 people. During the study period, all the SDI quintiles experienced a decline in the absolute burden of GC caused by a high-sodium diet (Table 1). The high-SDI quintile demonstrated the most substantial decrease in GC burden associated with elevated sodium consumption, with an EAPC of −2.72 (95% CI: −2.75 to −2.70) for mortality and an EAPC of −3.11 (95% CI: −3.13 to −3.08) for DALYs.

**Fig. 5:**
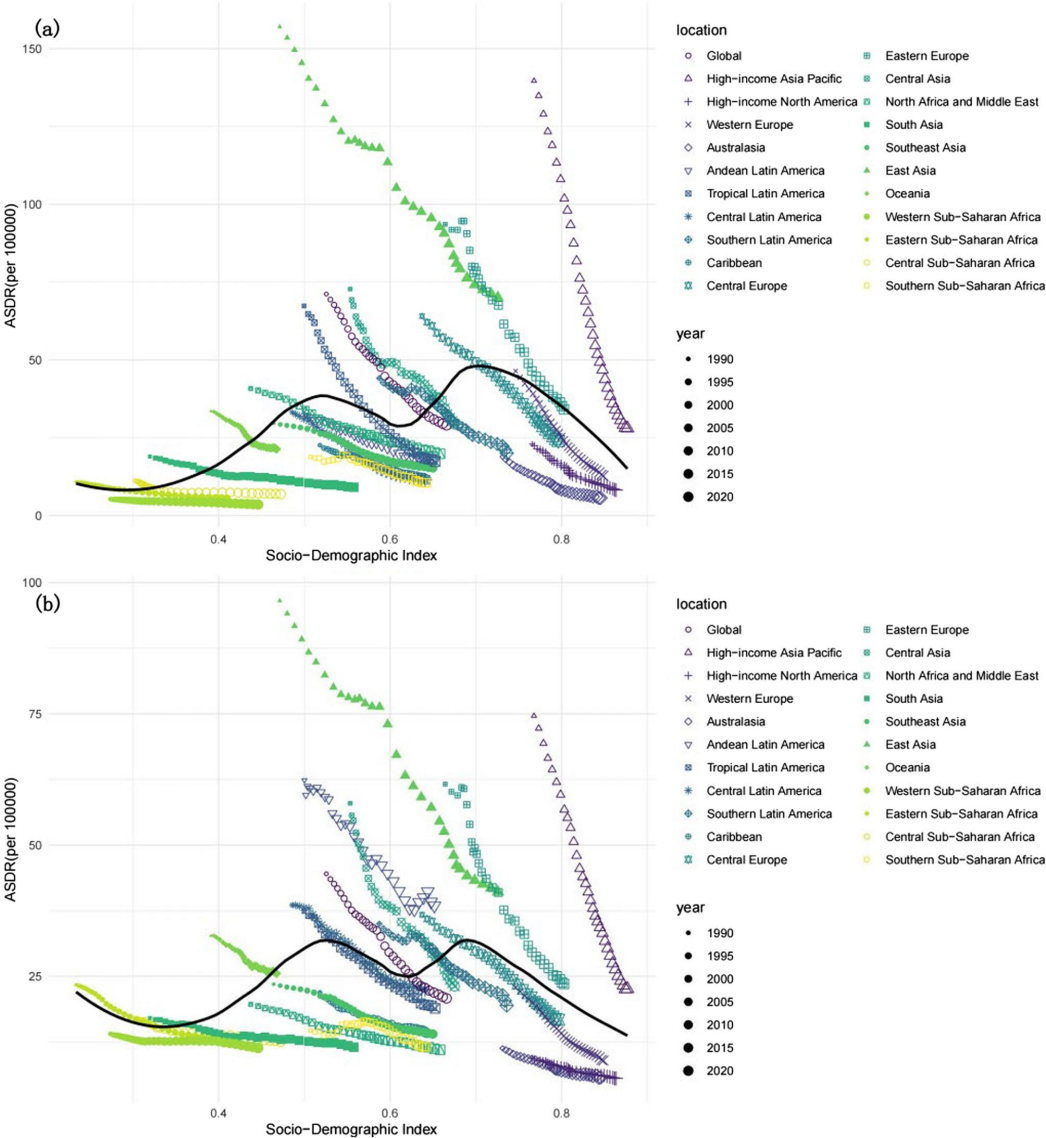
Age-standardised DALY rates (ASDR) of gastric cancer attributable to smoking (**a**) and a high-sodium diet (**b**) for 21 GBD regions by socio-demographic index, 1990–2021. Expected values based on socio-demographic Index and disease rates in all locations are shown as the black line

Figure 5a depicts the temporal trend of the DALY rates for the ASDR across the 21 regions, categorized by the SDI, from 1990 to 2021. Globally, the ASMRs and ASDRs caused by smoking show M-shaped correlations with the SDI. However, the ASMRs and ASDRs of GC in the 21 regions both tended to decrease. Notably, the ASDR in the high-income Asia Pacific region decreased significantly with increasing SDI values (Fig. 5a). From 1990 to 2021, East Asia and high-income North America displayed higher-than-expected ASMRs of GC attributed to smoking. Conversely, Australasia, Central Europe, Western Europe, Andean Latin America, Tropical Latin America, Southern Latin America, Southern Sub-Saharan Africa, and Western Sub-Saharan Africa presented lower-than-expected ASDR values for GC associated with smoking during the same period (Fig. 5). A comparable relationship was observed between the ASMR and the SDI (Fig. S1).

The long-term trends in the ASMRs and ASDRs for GC attributable to a high-sodium diet across SDI quintile categories by geographical area from 1990 to 2021 are shown in Fig. 5b and Fig. S2, respectively. With the increase in the SDI value, significant decreases in the ASMR and ASDR were observed in the high-income Asia Pacific and Tropical Latin America, whereas a more gradual decline was noted in South Asia and Western Sub-Saharan Africa. In East Asia, the ASMR and ASDR first decreased but subsequently slowly increased, reaching a peak SDI of 0.6, and then significantly decreased as the SDI value continued to rise (Fig. 5c and d).

Overall, in areas with a lower SDI, smoking and a high-sodium diet had greater effects on the burden of gastric cancer. From 1990 to 2021, a downwards trend in the ASMRs and ASDRs was observed across all the regions, albeit to varying degrees. In particular, the regions with high SDIs experienced the greatest declines, while the regions with low SDIs experienced smaller declines.

## Discussion

This study systematically examined the latest estimates of temporal trends in deaths and DALY burden of GC attributable to smoking and a high-sodium diet from 1990 to 2021 at the global, regional, and national levels, utilizing data from the GBD 2021 study. The findings revealed that the numbers of deaths, DALYs, and both ASMRs and ASDRs associated with GC decreased globally between 1990 and 2021, whereas the number of deaths from GC attributable to a high-sodium diet increased and the number of deaths attributable to smoking decreased. However, the burden of GC continues to exhibit substantial geographic variation across regions and countries. Additionally, the numbers of deaths and DALYs varied across age groups, with males having significantly higher numbers in each age group compared to females. Moreover, both the ASMRs and the ASDRs increased with age. Therefore, targeted and sustained efforts are necessary to reduce the deaths and DALY burden of GC attributable to smoking and a high-sodium diet.

The data we studied show that the proportions of gastric cancer deaths and DALYs attributed to smoking and a high-sodium diet are highest in the 65–69 and 70–74 age groups, which suggests that the disease persists from youth into middle and older adulthood, with the latter demographic facing increased risk due to a gradual decline in bodily functions and immune efficiency. Consequently, elderly people are particularly vulnerable to the negative effects of a high-sodium diet [19]. With the continuous advancement of modern precision treatment methods, such as targeted therapy, immunotherapy, and the widespread adoption of standardized surgery, the survival rate of patients with GC is gradually improving [20–23]. However, the number of cases among young patients is on the rise, with the disease often being more insidious in terms of onset and rapid progression [24]. This trend among younger patients is closely related to the lifestyles and dietary habits of younger generations. Our research, which focuses on the burden of GC attributable to smoking and a high-sodium diet, is therefore highly necessary.

Our analysis of the GBD data revealed that the burden of GC caused by smoking and a high-sodium diet is much greater in men than in women. Both the ASMRs and the ASDRs were significantly greater in men. This can be explained by several factors. First, the prevalence of smoking is significantly higher in men than in women, with some studies showing that the global daily smoking rate for men has been approximately 25% since 1990, compared with only 5.4% for women [25], and that smoking is more prevalent and accepted in men because of sociocultural influences, whereas smoking in women is subject to more social constraints [26]. It has also been shown that the gastric mucosa of men may be more sensitive to carcinogens in tobacco and that there is a synergistic effect between smoking and Helicobacter pylori infection, further increasing the risk of gastric cancer [27]. In addition, men usually consume more high-sodium foods, especially pickled foods and processed meats, which not only have high sodium content, but also may contain carcinogens such as nitrites, which further increase the risk of gastric cancer [28]; second, a high-sodium diet may promote colonization by H. pylori, and men may be more susceptible to infection with H. pylori because of their dietary habits and physiological characteristics [29]; In addition, men are more likely to smoke and consume alcohol at the same time, both of which synergize with a high-sodium diet to further increase the risk of GC [30]. Public health strategies must incorporate targeted interventions for male populations, integrating health education on balanced diets with behavioural management programs.

Over the past 30 years, the global burden of GC attributable to smoking has significantly decreased. From 1990 to 2021, both the ASMR and the DALY rate of GC due to smoking tended to decrease globally [13]. However, there were significant regional differences in this decline. Since the implementation and endorsement of the WHO Framework Convention on Tobacco Control in 2005, the smoking rate in many countries and regions around the world has continued to decline [31], therefore, in the past decade, the impact of smoking on the global burden of gastric cancer has been mitigated. Our study revealed that in East Asia, the impact of smoking on the ASMR and ASDR of patients with gastric cancer is significantly greater than in other regions.

In addition, the “M-shaped correlation” between smoking-related gastric cancer burden and the SDI reflects complex regional variations in risk accumulation and public health responses. East Asia’s higher-than-expected burden results from the persistent legacy of high-sodium diets and delayed tobacco control, demonstrating how historical exposures shape current disease patterns through cohort effects. Conversely, high-income Western regions have a lower burden because of the early implementation of tobacco control and Helicobacter pylori management. Moreover, low-SDI regions exhibit apparent under-ascertainment because of their limited diagnostic capacity and compressed age structure. These systematic deviations demonstrate that socioeconomic development alone cannot predict disease burden without considering (1) the timing of public health interventions, (2) historical dietary patterns, or (3) data quality. This explains why similar development levels produce different health outcomes, emphasizing the need for contextualized policy approaches beyond SDI-based projections.

Notably, for estimates of the gastric cancer burden linked to high-sodium intake, the absolute numbers of deaths and DALYs continue to increase even when the ASMRs and ASDRs decrease. This divergence is driven by two overarching demographic forces: rapid global population growth increases the pool at risk, and widespread population ageing increases the proportion of older adults, who carry the highest intrinsic gastric cancer risk. Because they remove the confounding effect of changing age structure, the standardized rates provide a clearer picture of the underlying temporal trend in risk factor impact, whereas the counts simply capture the net outcome of both risk factor change and demographic expansion.

Moreover, there are significant differences in the burden of GC caused by a high-sodium diet across different regions and countries. A high-sodium diet can directly damage the gastric mucosa and induce hyperplasia of the gastric fossa epithelium, thereby increasing the possibility of endogenous mutations. Furthermore, high sodium intake is associated with the progression of intestinal metaplasia, which is a known precancerous lesion of GC [32]. Previous studies have also shown that excessive sodium intake may change the thickness of the defensive mucus layer and promote colonization by Helicobacter pylori [33]. In East Asia, the greatest number of gastric cancer deaths are attributed to high-salt diets and excessive intake of pickled foods, with China being the greatest contributor. As China’s economy has developed rapidly, people’s dietary habits have changed significantly, characterized by increased consumption of high-salt and high-fat foods and decreased intake of grains and vegetables [34]. However, in the past few decades, dietary patterns in some high-income countries have remained relatively stable, with gastric cancer incidence rates also remaining relatively stable [35].

An analysis of SDI regions revealed the complex nature of the GC burden linked to smoking and a high-sodium diet. Overall, low-SDI areas are most strongly affected by smoking and high-sodium diets. Between 1990 and 2021, age-standardized mortality rates declined across all regions, but the magnitude of the reduction was clearly graded by development level: high-SDI populations experienced the sharpest decreases, whereas low-SDI populations showed only modest improvements. This disparity is driven largely by socioeconomic imbalances, underinvestment in health systems, and lower health literacy. Building on these findings, we stratified evidence-based salt-reduction and tobacco-control actions by SDI tier (Table 2), offering policy-makers a ready-to-use menu of tailored interventions to narrow the unequal distribution of gastric cancer worldwide.

**Table 2:** SDI-Stratified Actions for Salt Reduction and Tobacco Control to Mitigate GC Burden

SDI Tier	Salt Reduction Actions	Tobacco Control Actions
Low SDI	1. Promote low-cost, low-sodium cooking methods (e.g., using natural spices instead of salt) and conduct "salt-reduced cooking training" in communities; 2. Collaborate with local food producers to develop simple salt reduction standards for common processed foods (e.g., pickled products); 3. Disseminate knowledge on "GC risk from high-sodium diet" via public health posters and rural radio broadcasts.	1. Launch targeted tobacco hazard awareness campaigns (e.g., setting up anti-tobacco billboards in markets and health centers), with a focus on male populations; 2. Advocate for local governments to issue "public place smoking bans," prioritizing implementation in key areas like schools and hospitals; 3. Provide low-cost smoking cessation counseling services (e.g., establishing quit clinics in primary health centers).
Low-Middle/Middle-High SDI	1. Develop mandatory local standards for sodium content in foods (e.g., upper limits for soy sauce and snacks) and promote "sodium content warnings" on food packaging; 2. Encourage catering businesses to offer "low-sodium menus" and provide policy subsidies to those proactively adopting salt reduction options;3. Integrate "salt reduction knowledge" into primary and secondary school health education curricula to foster long-term healthy eating habits.	1. Increase tobacco tax rates to reduce tobacco consumption through fiscal leverage, and allocate part of the tax revenue to GC early screening programs; 2. Strengthen supervision of tobacco advertising, prohibiting tobacco promotions on social media and outdoor billboards; 3. Launch "smoke-free workplace/community" initiatives with incentives for smoking cessation (e.g., preferential health check-ups).
High SDI	1. Promote technological upgrades in the food industry to develop low-sodium, high-flavor alternative products (e.g., low-sodium soy sauce, low-salt seasonings) to minimize taste impact of salt reduction; 2. Provide "personalized salt reduction guidance" for high-risk groups (e.g., adults aged ≥65 years) and assess salt reduction effects through regular health check-ups; 3. Enhance cross-regional collaboration to learn from experiences of "combined salt reduction and H. pylori eradication interventions" in high-SDI East Asian regions.	1. Consolidate existing tobacco control achievements, such as expanding e-cigarette regulation and increasing the font size of health warnings on tobacco products; 2. Address the "rising smoking rate among young people" through youth-oriented anti-tobacco campaigns on short-video platforms and social media; 3. Include smoking history as an indicator in GC early screening risk assessments, prioritizing endoscopy for long-term smokers.

Strengths of this study include comprehensive, up-to-date estimates of the situation and trends of gastric-cancer burden attributable to smoking and a high-sodium diet over 1990–2021 using GBD 2021. Several limitations merit emphasis. First, it is noteworthy that the 95% uncertainty intervals for the burden attributable to high-sodium diet were often wide and included zero, reflecting conservative priors and sparse data within the Bayesian framework. These intervals accurately depict the range of values compatible with existing evidence but highlight low precision—an inherent limitation underscoring the urgent need for standardized dietary-surveillance systems and region-specific epidemiological studies. Consequently, while point estimates support continued salt-reduction policies, the wide UIs hinder accurate forecasting of intervention benefits in data-sparse regions. Second, estimates for many less-developed countries relied on predictive covariates or neighbouring trends because of absent cancer registries, potentially introducing bias. Third, heterogeneity across study designs, populations and data-collection methods may affect risk-factor estimates; although GBD modelling mitigates this, some residual bias may persist. Fourth, we examined only smoking and high sodium, ignoring H. pylori infection, genetic factors and other environmental determinants. Finally, spatiotemporal analyses for alcohol or red- and processed-meat diets were precluded by the lack of corresponding GBD data.

## Conclusion

Although the ASMRs and ASDRs of GC attributable to smoking and a high-sodium diet have decreased globally, owing to population growth and ageing, the absolute number of deaths caused by a high-sodium diet for gastric cancer continues to increase, and there are still significant regional differences among different age groups and regions. Notably, East Asia, especially China, had the greatest number of GC-related deaths and DALYs attributable to smoking and a high-sodium diet. Our research focused on identifying two specific modifiable risk factors—smoking and a high-sodium diet—associated with the burden of GC across various geographical levels, aiming to highlight these crucial factors for the prevention and control of GC, thereby contributing to the achievement of Sustainable Development Goals 3.4.

## Supplementary Information

Supplementary Material 1.

## Data Availability

The datasets generated and/or analyzed during the current study are available in the Global Burden of Disease Study repository (http://ghdx.healthdata.org/gbd-results-tool), accessed on 6 March 2025. The specific query parameters are detailed in Supplementary Table 6. Additional data are available from the corresponding author upon reasonable request.
