# Supplementary material for: Global, regional, and national burden of gastric cancer attributable to smoking and a high-sodium diet from 1990 to 2021: a global burden of disease study 2021
Source: BMC Gastroenterol. 2025 Dec 3;26:52. doi: 10.1186/s12876-025-04431-8 (PMC12828996; doi:10.1186/s12876-025-04431-8)
Supplement: Supplementary file 1 — Supplementary Material 1. [file 12876_2025_4431_MOESM1_ESM.zip › Supplementary Information/Supplementary Information/Data Query and Detailed Information Retrieval.docx]

**Data Query and Detailed Information Retrieval:**

| Tool | GBD Results Tool (https://ghdx.healthdata.org/gbd-results-tool) |
| --- | --- |
| GBD Estimate | Risk factor |
| Measure | Deaths,DALYs |
| Metric | Number,Rate |
| Risk | Smoking (ID:99),Diet high in sodium (ID:124) |
| Cause | Stomach cancer (ID:414) |
| Location | Global,Low SDI, Low-middle SDI, Middle SDI, Middle-high SDI, High SDI, 21 regions, 204 countries |
| Age | All ages & Age-standardized& 25-year bands (25-29, 30-35, ..., 95+) |
| Sex | Both,Male,Female |
| Year | 1990-2021 |
