# Supplementary material for: Global, regional, and national burden of gastric cancer attributable to smoking and a high-sodium diet from 1990 to 2021: a global burden of disease study 2021
Source: BMC Gastroenterol. 2025 Dec 3;26:52. doi: 10.1186/s12876-025-04431-8 (PMC12828996; doi:10.1186/s12876-025-04431-8)
Supplement: Supplementary file 1 — Supplementary Material 1. [file 12876_2025_4431_MOESM1_ESM.zip › Supplementary Information/Supplementary Information/Supplementary Figure.pdf]

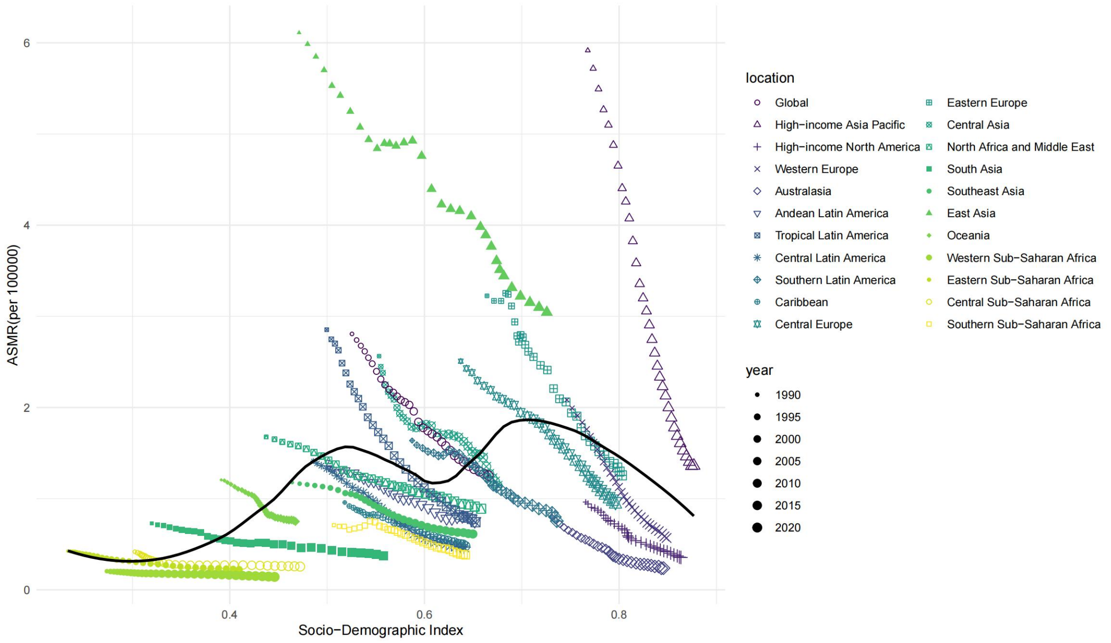

Supplementary Figure 6 Age-standardised mortality rates (ASMRs) of GC attributable to smoking for21 GBD regions by socio-demographic index, 1990-2021.

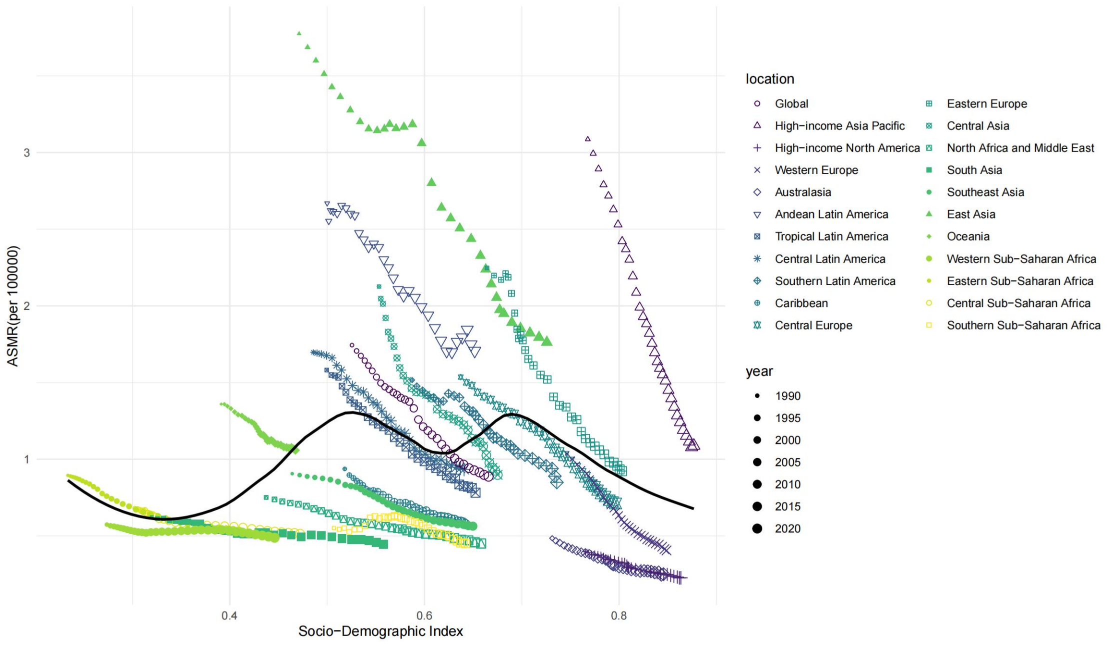

Supplementary Figure 7 Age-standardised mortality rates (ASMRs) of GC attributable to a high-sodium diet for 21 GBD regions by socio-demographic index, 1990-2021.
